# Empirical Retrieval of Surface Melt Magnitude from Coupled MODIS Optical and Thermal Measurements over the Greenland Ice Sheet during the 2001 Ablation Season

**DOI:** 10.3390/s8084915

**Published:** 2008-08-22

**Authors:** Derrick Lampkin, Rui Peng

**Affiliations:** Department of Geography, College of Earth and Mineral Sciences, Pennsylvania State University, USA; E-Mails: djl22@psu.edu (F. L.); rpx@psu.edu (F. L.)

**Keywords:** Greenland, remote sensing, surface melt

## Abstract

Accelerated ice flow near the equilibrium line of west-central Greenland Ice Sheet (GIS) has been attributed to an increase in infiltrated surface melt water as a response to climate warming. The assessment of surface melting events must be more than the detection of melt onset or extent. Retrieval of surface melt magnitude is necessary to improve understanding of ice sheet flow and surface melt coupling. In this paper, we report on a new technique to quantify the magnitude of surface melt. Cloud-free dates of June 10, July 5, 7, 9, and 11, 2001 Moderate Resolution Imaging Spectroradiometer (MODIS) daily reflectance Band 5 (1.230-1.250μm) and surface temperature images rescaled to 1km over western Greenland were used in the retrieval algorithm. An optical-thermal feature space partitioned as a function of melt magnitude was derived using a one-dimensional thermal snowmelt model (SNTHERM89). SNTHERM89 was forced by hourly meteorological data from the Greenland Climate Network (GC-Net) at reference sites spanning dry snow, percolation, and wet snow zones in the Jakobshavn drainage basin in western GIS. Melt magnitude or effective melt (E-melt) was derived for satellite composite periods covering May, June, and July displaying low fractions (0-1%) at elevations greater than 2500m and fractions at or greater than 15% at elevations lower than 1000m assessed for only the upper 5 cm of the snow surface. Validation of E-melt involved comparison of intensity to dry and wet zones determined from QSCAT backscatter. Higher intensities (> 8%) were distributed in wet snow zones, while lower intensities were grouped in dry zones at a first order accuracy of ∼ ±2%.

## 1. Introduction

The changing masses of Greenland and Antarctica represent the largest unknown in predictions of global sea-level rise over the coming decades (Dowdeswell, 2006). Recent analysis suggests that the contribution of the Greenland and Antarctic Ice Sheets to present-day sea level rise is more than 0.3 millimeters per year (Krabill et al., 2000; Rignot and Thomas, 2002; Shepherd and Wingham, 2007). Changes in surface temperature on these large ice masses can affect the rate of ice deformation or basal sliding (Zwally et al., 2002). Rapid increases in the extent and duration of surface melt have been detected using satellite imagery along the surface of southern Greenland and parts of Antarctica (Jezek et al., 1992; Ridley, 1993; Zwally and Fiegles, 1994; Abdalati and Steffen, 1995; Mote and Anderson, 1995, and several others.) Zwally et al. (2002) have demonstrated that ice flow speed increases during the summer melt season. Rignot and Kanagaratnam (2006) have confirmed acceleration of ice flow over a large part of coastal Greenland between 1996 and 2000 from radar interferometry and attribute this to recent climate warming.

In recent years, observations of ice sheet physical properties and dynamic behavior have shifted from in-situ observations towards satellite techniques (Lubin and Masson, 2006). The ability of satellite systems to acquire data over vast areas of remote terrain, during the day or night and in all weather conditions has facilitated this shift (IGOS, 2007). A variety of satellite instruments sensitive to different parts of the electromagnetic (EM) spectrum provide rich data sets of elevation, motion, accumulation on ice sheets (Bindschadler, 1998). Understanding the mechanism and features of the current approaches of modeling of ice sheet melt is critical to improve the assessment of melt dynamics over Greenland Ice Sheet (GIS). This work demonstrates a novel approach to improve the assessment of melt dynamics by expanding satellite derived estimates of melt from a binary measure of occurrence to melt magnitude or intensity.

## 2. Background

Snow is a mixture of ice, liquid water and air. The dielectric constant of snow is derived from a weighted average of the dielectric constants of these components (Matzler et al, 1984). The real part of the dielectric constant of ice has a value of 3.17 throughout the microwave region. Specifically, microwave emission in dry snow is dominated by scattering (Cumming, 1952; Tiuri et al., 1984; Rees, 2006). Consequently, liquid water in wet snow increases the dielectric constant of snow and thus enhances emissivity and absorption of microwave radiation (Chang et al., 1976; Tiuri et al., 1984; Hallikainen, 1986). Given these relationships between microwave emission and ice properties, airborne and satellite based systems have been used successfully to map ice properties and assess surface and near-surface melt conditions. Active radar systems such as Synthetic Aperture Radar (SAR) provide high-resolution observations of microwave radar backscatter, and have been applied to study GIS surface and near-surface properties and ice dynamics (Fahnestock et al., 1993). Ranging radars operating at 5.3 GHz and 13.3 GHz have been used and compared with in-situ data by Jezek et al. (1994) to interpret melt-related processes on the GIS. NASA Scatterometer data (NSCAT) were combined with Seasat Scatterometer (SASS) data and ERS-1/2 Scatterometer data (ESCAT) to map ice sheet melt extent (Long and Drinkwater, 1999). Due to its ability to penetrate into snow, normalized radar cross section (NRCS) measurements from C-band (5.3 GHz) scatterometers were used to monitor seasonal snowmelt on Greenland by Wismann (2000).

Rapid increases in the extent and duration of surface melt on the GIS from 1978 to the present have been detected from passive microwave systems such as the Scanning Multi-channel Microwave Radiometer (SMMR) and the Special Sensor Microwave/Imager (SSM/I) deployed on several Defense Meteorological Satellite platforms (http://cires.colorado.edu/steffen/).

The presence of liquid water has a dramatic effect on the microwave properties of snow. The Rayleigh-Jeans approximation for radiation in the microwave part of the EM spectrum is

(1) $$T_{b}\left(\lambda \right)=\varepsilon T$$

where T_b_ is the brightness temperature, *ε* is the microwave emissivity, and T is the effective physical temperature of the snow (Abdalati and Steffen, 1997). Compared with dry snow, whose dielectric constant is a function of density only in the microwave region, the dielectric behavior of wet snow is a function of its physical parameters and frequency (Hallikainen et al., 1986). Therefore, according to the Rayleigh-Jeans approximation, the emissivity is relatively constant over time for dry snow, in which case the T_b_ is approximately a linear function of T. When melt occurs, liquid water causes a large increase in the emissivity of snow and results in a corresponding increase in T_b_ (Ashcraft and Long, 2003).

Changes in T_b_ at 19 and 37 GHz have been used as a metric for determining melt onset (Zwally and Fiegles, 1994; Ridley, 1993, and Mote and Anderson, 1995). Steffen et al., (1993) identified wet snow regions using AVHRR (Advanced Very High Resolution Radiometer), SMMR, SSM/I and in-situ data, based on the relationships between in-situ measurements and horizontally polarized 19 and 37 GHz observation. Specifically, the cross-polarization gradient ratio (XPGR) (Abdalati and Steffen, 1995) approach was used to assess melt zones. XPGR indicates melt when the snow surface contains greater than 1% liquid water by volume. To study seasonal and inter-annual variations in snow melt extent of the ice sheet, Abdalati and Steffen (1997) established melt thresholds in the XPGR by comparing passive microwave satellite data to field observations. Ashcraft and Long (2005) studied the differentiation between melt and freeze stages of the melt cycle using the SSM/I channel ratios. In 2006, these authors assessed melt detection performance from SSM/I, SeaWinds on QuikSCAT (QSCAT), and the European Remote Sensing (ERS) Advanced Microwave Instrument (AMI) in scatterometer mode, and concluded that melt estimates from different sensors were highly correlated. The difference between ascending and descending brightness temperatures (DAV) (Ramage and Isacks, 2002) measured either at 19.35- or 37- GHz by SSM/I was applied to map melt extent in Greenland, and the results compared with those obtained from QSCAT (Nghiem et al., 2001; Tedesco, 2007).

Although active and passive microwave systems have performed well in monitoring melt conditions over the GIS, they are limited in the amount of detail that can be either spatially or temporally resolved. Passive systems have relatively coarse spatial resolution and generally results from maintaining high radiometric resolution, while active systems demonstrate limited or lower temporal resolution (Campbell, 2007). Active systems such as SAR in high-resolution observations of microwave radar backscatter have 16-day ground track repeat cycle, which is too infrequent to capture dynamic melt conditions.

Other parts of the EM spectrum offer potential advantages for monitoring melt over the GIS, and may augment the shortcomings of microwave systems. Data from optical satellites have been used to map surface dynamics related to the melt process over the GIS at higher spatial resolutions. Hall et al. (1990) compared in-situ measurements with Landsat Thematic Mapper (TM)-derived reflectance on Greenland and concluded that Landsat TM was viable to obtain the physical reflectance of snow and ice. AVHRR visible and near-infrared radiances were used to derive surface albedo over the GIS and were validated by in-situ data (Stroeve et al., 1997). Products from the Moderate Resolution Imaging Spectroradiometer (MODIS) were also widely used to retrieve snow albedo over Greenland, and have been compared with in-situ measurements and with other instruments such as Multi-angle Imaging SpectroRadiometer (MISR) separately (Stroeve and Nolin, 2002a; Stroeve et al., 2004). Stroeve and Nolin (2002b) developed two different methods to derive the snow albedo over the GIS: one utilizing the spectral information from MISR and one based on angular information from the MISR instrument. Their results indicated that the accuracy of either of those two methods was within 6% compared of in-situ measurements. Hall et al. (2004) compared MODIS with SSM/I-derived melt extent in summer of 2002 and concluded that the results are not, and should not necessarily be, the same. They also suggested that MODIS and SSM/I data were complementary in providing detailed information about the maximum snow melt on the GIS.

Satellite derived thermal information over GIS has been addressed over the past two decades (Key and Haefliger, 1992; Haefliger et al., 1993; Comiso et al., 2003 and others). Specifically, Hall et al. (2006) examined mean clear-sky MODIS derived surface temperature over GIS from 200 to 2005 during the melt season and have determined that during periods of intense melting, surface temperatures were highest in 2002 (-8.29 ± 5.29°C) and 2005 (-8.29 ± 5.43°C) relative to the 6-year mean (-9.04 ± 5.59°C). More Recently, Hall et al. (2008) assessed the relationship between ice sheet mass balance and the spatio-temporal variability of MODIS retrieved surface temperature over GIS from 2000 to 2006. Daily, clear-sky MODIS land-surface temperature (LST) was compared to changes in mass concentration derived from the Gravity Recovery and Climate Experiment (GRACE) system and assessed that a mean LST increase (∼0.27°C per year) over this period was associated with an increase in melt season length and rapid mass loss during significant warming events, particularly at elevations below 2000 meters in 2004 and 2005.

Surface melt patterns and their duration are an important component of ice sheet mass balance, and have been successfully measured, however estimation of ice sheet surface melt amount is still underdetermined from passive microwave approaches. The missing link in improved modeling of ice sheet response to an increase in temperature is a full assessment of surface melt amount.

### 2.1. Optical and Thermal Radiative Theory

Surface albedo, which influences the amount of absorbed solar radiation, can vary due to several factors such as grain-size, emission angle, snow density, surface impurities, and liquid water content (Dozier and Warren, 1982). Previous work has examined the strong relationship between snow spectral reflectance and grain-size, and modeled snow reflectance from the optical properties of snow and ice (Bohren and Barkstrom, 1974; Wiscombe and Warren, 1981; Nolin and Dozier, 2000; Painter et al., 2003). The optical properties of snow indicate high reflectance in the visible (0.4-0.7μm), and parts of the Near Infrared (NIR) (0.7-1.3μm) regions of the EM spectrum. Snow reflectance demonstrates substantial decrease in the Shortwave Infrared (SWIR) (1.3-3μm) due to increase in absorption.

In the visible and NIR regions of EM spectrum, the optical properties of snow depend, in large part, on the refractive index of ice (Dozier, 1989). Absorption is due to variation in the imaginary part of the complex refractive index of ice given as:

(1) ε=n+ik

where *n* = real part of the refractive index, *k* = imaginary part of refractive index. The absorption coefficient (i.e., the imaginary part of the refractive index) varies substantially in the wavelengths from 0.4 to 2.4 um. In the near-infrared region of EM spectrum, the reflectance of wet snow is lower than that of dry snow, but mainly because of micro-structural changes caused by the water (Dozier, 1989).

Specifically, in wet snow with high liquid water content, heat flow from large grains causes smaller particles, which are at lower temperature, to melt and merge into larger clusters (Colbeck, 1982; Colbeck, 1989). As bulk grain cluster radius increases, an incident photon will have a high probability of being scattered when it transverses the air-ice interface, but a greater chance of absorption while passing through the ice grain (Warren, 1982). Grain clusters optically behave as single grains, increasing the mean photon path length, subsequently increasing the opportunity for absorption and reduction in reflectance. Larger grains increase the degree of absorption, particularly in the shortwave infrared region, causing a substantial reduction in reflectance. The maximum sensitivity of reflectance to changes in grain size is in the shortwave (SWIR) region of the EM spectrum at approximately 1.1 um (Nolin and Dozier, 2000).

The use of snow surface reflectance alone to track the surface melt process is not sufficient, because substantial decreases in reflectance are not due solely to grain enlargement associated with entrained liquid water. For example, small amounts of absorbing impurities can also reduce snow reflectance in the visible wavelength (Warren and Wiscombe, 1981). As low as 0.1 ppmw (parts per million by weight) of soot concentrations are enough to reduce reflectance perceptibly, and the effect is significantly enhanced when the impurities are inside the snow grains, because refraction focuses the light on the absorber (Grenfell et al., 1981; Bohren, 1986). In this case, surface temperature can be used as a plausible mechanism in isolating the component in reduced reflectance that is due to the melt process.

Snow-cover melt dynamics in the thermal infrared region (8-14um) of the EM spectrum are a function of incident radiation as well as surface longwave emission, which is a function of snow surface temperature and emissivity (Marks and Dozier, 1992). The relationship is given as follows:

(2) $$T_{b}(\lambda ,\mu_{v})=\frac{\text{hc}}{k\lambda \ln\left[\frac{e^{\text{hc}/(k\lambda T)}+\varepsilon -1}{\varepsilon }\right]}$$

where T_b_ is brightness temperature, which is defined as the temperature of a blackbody for a given wavelength that emits the same amount of radiation at that wavelength as does the snow; T is surface temperature; *ε* is the emissivity of snow; λ is wavelength; and h, c, k are constants(Dozier and Warren, 1982).

Therefore, the combination of optical and thermal signatures is an effective way to monitor the evolution of surface melt dynamics. Lampkin and Yool (2004a) have evaluated MODIS visible and NIR bands for monitoring snowpack ripeness and suggested that couple optical/thermal measurements have the potential to detect snowpack evolution during the melt season. Furthermore, Lampkin and Yool (2004b) have assessed surface snowmelt by developing a near-surface moisture index (NSMI) that uses optical and thermal variables.

## 3. Methods

### 3.1 Data

MODIS 8-day composite, 1 km^2^ resolution Land Surface Temperature (LST) (MOD11A2) version 5 (Wan et al., 2002) and 500m resolution Surface Reflectance (MOD09A1) version 5 (Vermote et al., 2002) products were used in this project. The primary advantage using optical and thermal measurements over passive microwave is the enhanced spatial resolution, while a major disadvantage is the reduced spatial coverage due to clouds. The effect of cloud cover is a major source of noise due to similar radiative behavior of clouds and snow in VIS, NIR, and SWIR regions of EM spectrum except at around 1.6 um. Generally, snow is a collection of ice grains, air, and liquid water, and often includes particulate and chemical impurities. Similarly, clouds contain water droplets, ice crystals and usually some impurities (Dozier, 1989). This similarity makes it difficult to distinguish snow and cloud in those regions of the EM spectrum. In addition, persistent cloud cover over Greenland is a severe limitation to full coverage daily acquisitions (Klein and Stroeve, 2002). The 8-day composite products were selected because they have fewer cloud cover than daily images, and increase coverage of the study area. MOD09A1 provides Bands 1-7 in an 8-day gridded level-3 product. Each MOD09A1 pixel was selected based on high observation coverage, low sensor view angle, the absence of clouds or cloud shadowing, and aerosol loading, producing the best possible daily observation during an 8-day period (http://edcdaac.usgs.gov/modis/mod09a1v5.asp). MOD11A2 were composited from the daily 1km LST product and stored on a 1km grid as the average values of clear-sky LST during an 8-day period (http://edcdaac.usgs.gov/modis/mod11a2v5.asp). There are a total of three 8-day composite scenes during the study period, spanning the period May 25 to June 17, 2001. All MODIS products used in this analysis were acquired over tiles H15V02, H16V02, H16V01, and H17V01. There are two tiles (H16V00, H17V00) of reflectance data over the GIS missing for the entire period.

QSCAT backscatter data were used to derive an estimate of wet and dry firn regions as a relative validation of the melt magnitude retrieval algorithm. QSCAT Enhanced Resolution Image products have wide swath and frequent over- flights, which permit generation of a wide variety of products. QSCAT is a dual-pencil-beam conically scanning scatterometer with the outer beam V-pol and the inner beam H-pol (http://www.scp.byu.edu/data/Quikscat/SIR/Quikscat_sir.html). The 2.225 km QSCAT Greenland H-pol and V-pol all-pass products were used for the comparison due to their high spatial resolution. Local overpass times for QSCAT ascending and descending orbits were approximately 6am and 6pm. Nghiem et al. (2001) have mapped snowmelt regions on GIS using SeaWinds Ku-band (13.4GHz) scatterometer on the QSCAT satellite by thresholding the difference in day and night backscatter images. This method defines the dry-snow zone on the ice sheet when the diurnal backscatter change is less than 1.8dB, and the wet-snow zone when the diurnal backscatter change is larger than 1.8dB (Nghiem et al., 2001).

**Figure 1.**
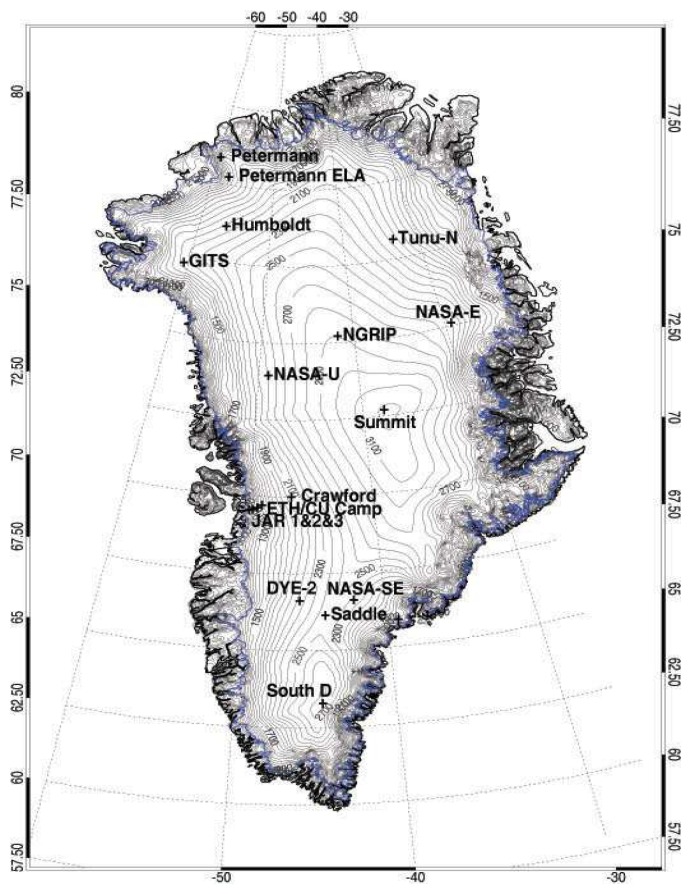
Location of stations in the Greenland Climate Network (GC-NET) as well as ice sheet elevation (Source: http://cires.colorado.edu/science/groups/steffen/gcnet/).

The Greenland Climate Network (GC-Net), established in 1995 monitors the climatology of GIS, and consisted of 18 Automatic Weather Stations (AWS) by 2001 (Figure 1) (Steffen and Box, 2001). Each AWS is equipped with a number of meteorological instruments (Table 1) that measure precipitation, incoming and outgoing shortwave and net radiation, air temperature, relative humidity, barometric pressure, wind speed and direction as well as snow pack temperature (Steffen and Box, 2001). Data from CP1 (Crawford Point 1), JAR1, and JAR2 stations were selected for this analyses because they span a range in elevation from 2022 to 568 meters, which represents a range of melt conditions from the accumulation to the ablation zones of the ice sheet (Table 2).

### 3.2 Model Development Scheme

**Figure 2.**
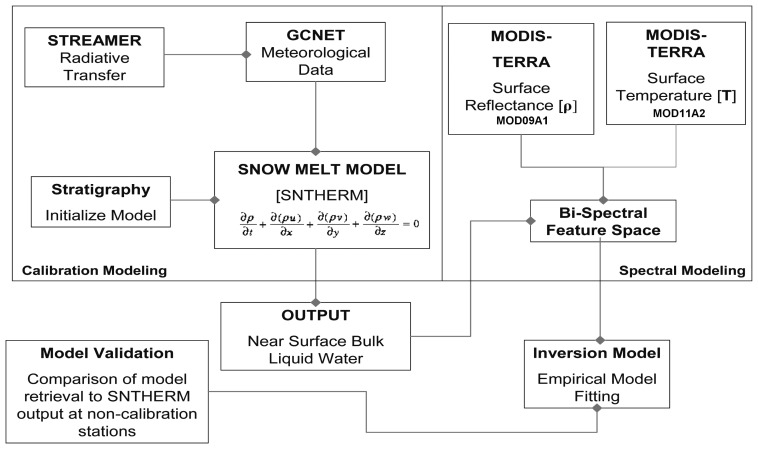
Algorithm Development Schematic depicting Calibration phase involving use of physical-based snowmelt model (SNTHERM89) initialized with stratigraphic data and forced by meteorological data. The Spectral modeling phase includes the construction of bi-spectral feature space from coupled shortwave infrared reflectance (1.230um < λ <1.250um) and surface temperature from MODIS.

Retrieval of melt magnitude is derived from coupled satellite surface reflectance and temperature observations, calibrated by model snowmelt estimates of liquid water content. Figure 2 depicts the algorithm development process, which is divided into the Calibration and Spectral modeling phases. The Calibration modeling phase produces estimates of snow pack near surface bulk liquid water content using the physical-based snowmelt model SNTHERM89. SNTHERM89 is a one-dimensional mass and energy balance model for estimating mass and energy flux through strata of snow and soil. It is comprehensive in scope, capable of simulating dynamic processes (Jordan, 1991). The version used in this project was adapted to estimate model snow melt conditions over glacier ice, which involved adding ice material properties to the SNTHERM89 material library (modifications courtesy of S. Frankenstein, CRREL). SNTHERM89 is initialized using snow pack stratigraphy and measured meteorological conditions over a given period, and computes mass and energy flux through the strata using a finite-difference scheme. SNTHERM89 divides the snow and underlying soil into *n* horizontally infinite plane-parallel control volumes of area A and variable thickness *Δz*. Generally the grid is constructed so that volume boundaries correspond to the natural layering of the snow cover, but the grid is allowed to compress as snow compacts over time (Jordan, 1991).

Cloud cover affects the net radiation balance to a large degree. Additionally, cloud-cover shifts the spectral distribution of incident radiation towards lower λ as a function of cloud absorption in the NIR spectrum (Grenfell and Maykut, 1977; Wiscombe and Warren, 1981). Therefore, assessment of cloud cover amount was necessary to be derived during the Calibration Phase. Streamer was used to derive an atmospheric effective opacity (O_e_) index, developed by Box (1997), as an indicator of percent radiative depletion of downwelling solar radiation by clouds. Streamer is a radiative transfer model which can be used for computing either radiances (intensities) or irradiance (fluxes) for a wide variety of atmospheric and surface conditions (Key and Schweiger, 1998). Effective opacity is given by:

(3) $$O_{e}=\frac{S↓-\text{DSRF}}{S↓}$$

where S↓ is theoretical clear-sky downwelling solar radiation flux, estimated from Streamer; DSRF is downwelling solar radiation flux measured from GC-NET stations. Effective opacity is a standardized measure ranging between 0 (completely clear conditions) to 1 for optically thick cloudy conditions (Box, 1997).

The Calibration Phase also involves preparation of meteorological data from Greenland Climate Network (GC-NET) stations to force SNTHERM89 over a test period spanning May 25 to June 17, 2001 melt season. This year was selected due to significant snow accumulation in the ablation zone within the last seven years (K. Steffen, personal communication, 2007).

Near surface bulk liquid water fraction (LWF), derived from SNTHERM89, was used to calibrate satellite derived optical-thermal signatures in the Spectral modeling phase. LWF is calculated by:

(4) $$\text{LWF}=\frac{<\text{bl}{>}_{8-\text{day}}}{<\text{bt}{>}_{8-\text{day}}}\times 100$$

where bl is Nodal Bulk Liquid Density (kg/m^3^), bt is Nodal Bulk Total Density (kg/m^3^). <bl> or <bt> is the 8-day average value of bl or bt for the upper 5 cm layers of snow focusing on the hours from 14:00 to 18:00, corresponding to MODIS local satellite overpass times. The liquid water fraction is only calculated from the upper 5 cm layers of snow, because 5 cm depth is the semi-infinite depth for optical bands, which means that an increase of snow depth beyond this value does not have any effect on the snow reflectance (Zhou et al., 2003). Though the effective depth for thermal emission is likely shallower than 5 cm (primarily < 1cm) (Dozier and Warren, 1982), the difference between liquid water fractions from SNTHERM89 less than 5 cm was not substantially different from those within 5 cm.

In this phase, MODIS 8-day composite, 500 meter reflectance at (1.230um <λ<1.250um) are rescaled to 1km and coupled with MODIS 1km 8-day composite surface temperature data. Reflectance data at this range (1.230um <λ<1.250um) is used because they are close to that part of the SWIR region where reflectance is highly sensitive to changes in grain size (Nolin and Dozier, 2000). Reflectance and temperature values were extracted from MODIS data at pixels corresponding to JAR1, JAR2, and CP1 stations (Table3). These pixels were extracted over three different composite periods 145 (May 25 -June 1), 153 (June 2 - June 9), and 161 (June 10 - June 17), in order to increase the number of samples upon which empirical linear models were to be created. Mean LWF was calculated for each site over the composite periods using Equation [5].

Satellite derived surface temperature and reflectance signatures were extracted from composited MODIS grids at the meteorological calibration sites (CP1, JAR1, and JAR2) for the 145, 153, and 161 composite periods (Table 3). A linear empirical model was developed with LWF as the dependent variable and MODIS extracted surface temperature and SWIR reflectance as independent variables (Table 4). This model was used to estimate surface melt magnitude or “effective” melt magnitude (E-melt) at 1 km^2^ spatial resolution across the GIS. For clarification, LWF is distinguished from E-melt as the magnitude of simulated bulk liquid water fraction estimated from SNTHERM89, while E-melt is a temporally integrated assessment of LWF fraction over 8-day composite periods retrieved from coupled MODIS optical/thermal signatures.

### 3.3 Sensitivity of SNTHERM89 to Initial Conditions

**Figure 3.**
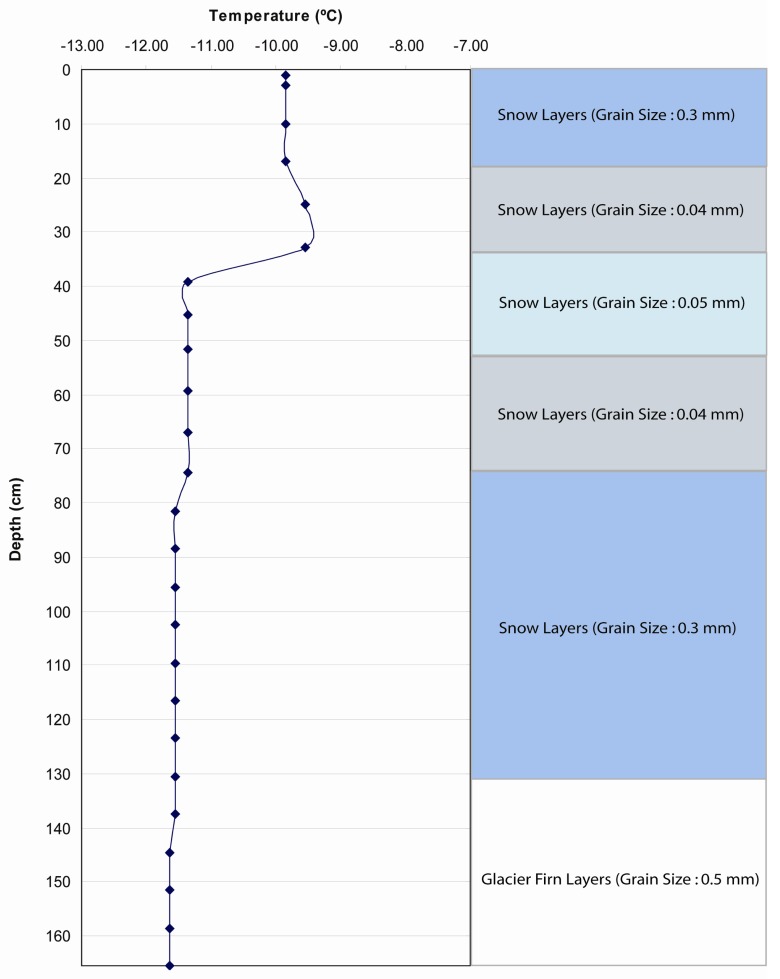
Snow pack and upper firn stratigraphy at Swiss camp excavated by K. Steffen on May 16, 2001.

Stratigraphy from Swiss Camp (ETH/CU) (Figure 3), courtesy of K. Steffen (CIRES/CU Boulder), excavated on May 16, 2001, was the only data available for this season in the study region. Therefore, SNTHERM89 was initialized at each calibration site (JAR1, JAR2, and CP1) using the same stratigraphy. It was assumed that estimated parameters derived from the snowmelt model will represent local meteorological conditions if the model was executed sufficiently ahead of the analysis period (May 25 to June 17, 2001). If this were true, then the use of stratigraphic data from ETH/CU camp would not bias the model output. This assumption was tested by comparing the difference between SNTHERM89 outputs derived from starting the model with stratigraphy from ETH/CU with model output initialized with several test stratigraphy. These stratigraphy sensitivity tests were executed using meteorological forcing data from two GC-NET stations that represent high (JAR1) and low (CP1) melt magnitude conditions, during the period from May 26 to Jun 16, when station data from these two sites overlap. Given that JAR1 lacked data from the period before May 26, a combined data set was created using meteorological data from ETH/CU. Because ETH/CU is the nearest station to JAR1, it is assumed that meteorological conditions at JAR1 were not much different from those at ETH/CU.

**Figure 4.**
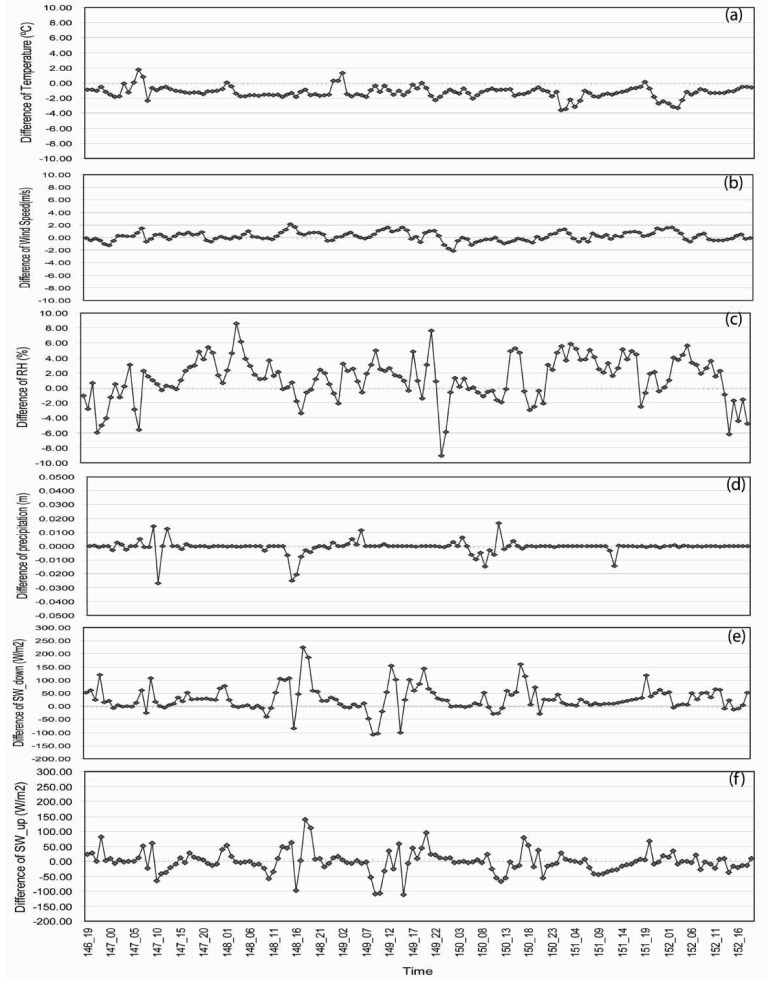
Difference between the SNTHERM89 input meteorological variables from JAR1 and ETH for the overlapping period from May 26 to Jun 01, 2001.(a) Temperature (°C); (b) Wind Speed (m/s); (c) Relative Humidity (RH) (%); (d) Precipitation (m); (e) SW_ down (W/m^2^); (f) SW_ up (W/m^2^).

Difference in forcing variables derived from these two station were examined in order to create a combined meteorological forcing data set from JAR1 and ETH/CU stations during an overlapping period (Figure 4). Data at these two stations demonstrated some short duration differences in wind speed, incident solar radiation (SW _ down), and upwelling solar radiation (SW _ up). Conversely, temperature, relative humidity, and precipitation did not exhibit significant differences.

**Figure 5.**
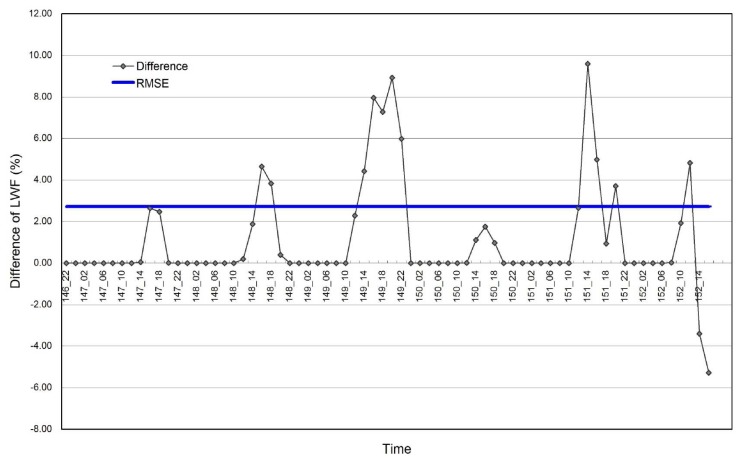
Comparison of estimated LWF at ETH\CU and JAR1 GCNET stations initialized with ETH stratigraphy from May 26 to Jun 01, 2001 demonstrating a RMSE of 2.72%.

Comparison of simulated LWF from SNTHERM89, forced with GC-NET data from ETH/CU and JAR1, and initialized with ETH/CU stratigraphy during the overlapping test period, demonstrated a Root Mean Square Error (RMSE) of 2.72% (Figure 5). This seems sufficiently low to support merging meteorological forcing data from ETH/CU and JAR1 to provide a seamless and uninterrupted time series.

**Figure 6.**
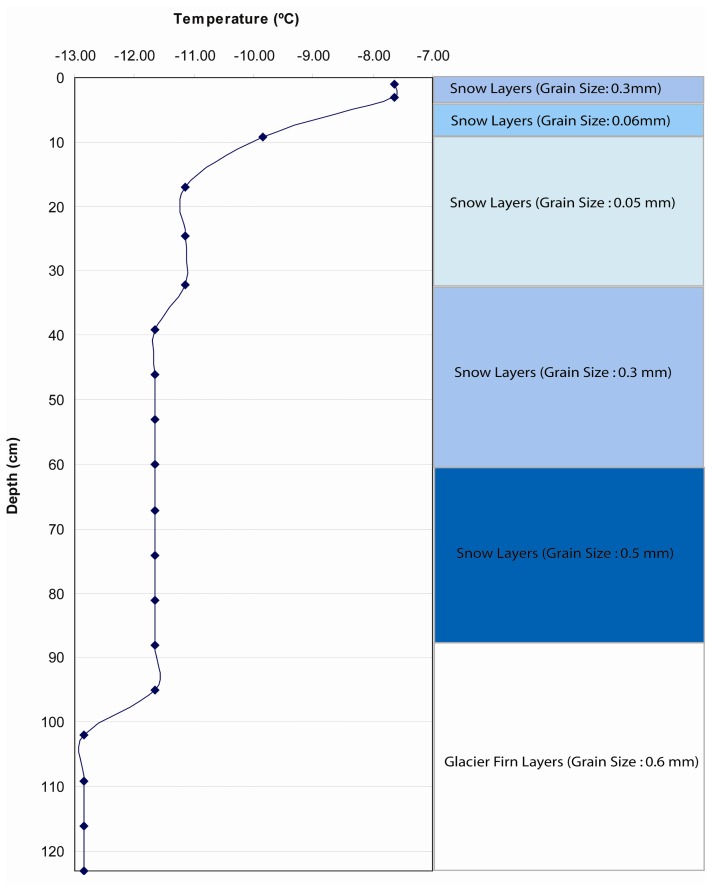
Fabricated test stratigraphy used to test SNTHERM89 temporal sensitivity to initial conditions in Test 1. Both temperature and grain size in this test stratigraphy were in a wider range than the ETH/CU stratigraphy.

Several test strata (Table 5) were designed by varying the values of temperature and grain size. Test 1 stratigraphic information (Figure 6) was designed to be different from the ETH/CU stratigraphy with larger variability of temperature and grain size than ETH/CU stratigraphy. Historic strata were used to evaluate a greater range in initialization modes. These strata were excavated by C. Benson (Benson, 1962) during the 1955 traverse, over 4 years.

**Figure 7.**
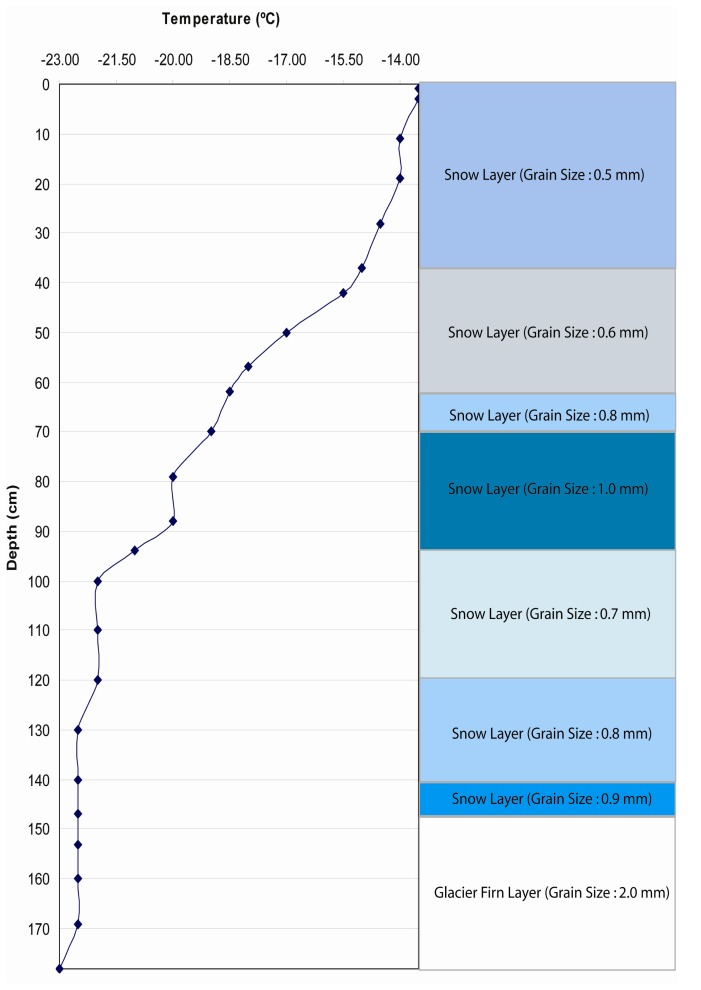
Snow pack and upper firn stratigraphy used to test SNTHERM89 temporal sensitivity to initial conditions in Test 2. This stratigraphy was excavated by C. Benson on May 17, 1955 at a site in northern Greenland with the elevation of 1310 meter.

**Figure 8.**
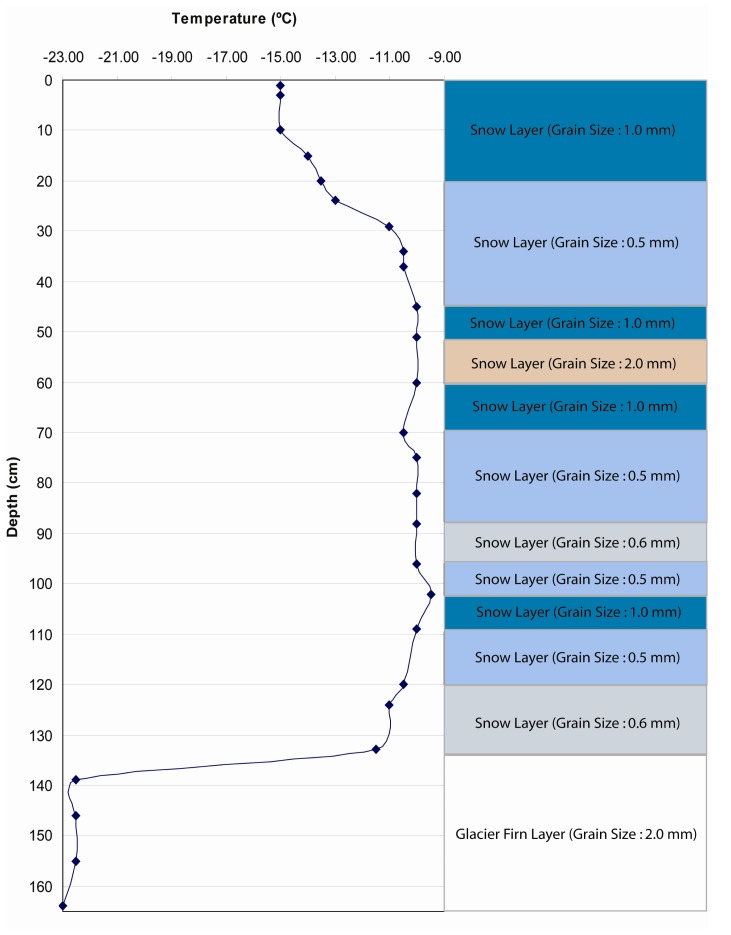
Snow pack and upper firn stratigraphy used to test SNTHERM89 temporal sensitivity to initial conditions in Test 3. This stratigraphy was excavated by C. Benson on August 18, 1955 at a site in Greenland inland with the elevation of 1963 meters.

**Figure 9.**
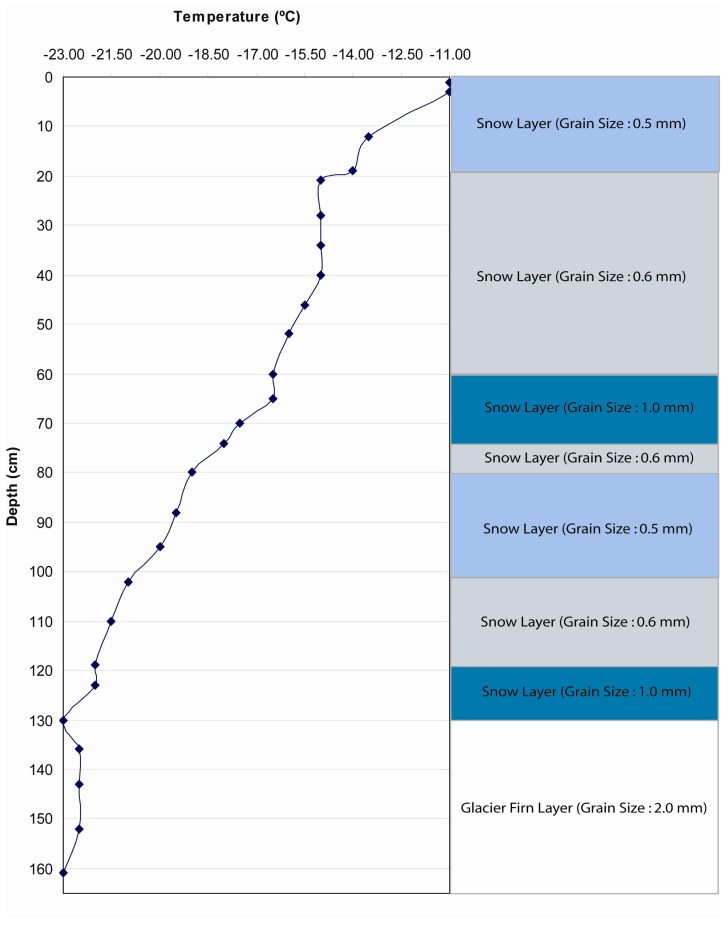
Snow pack and upper firn stratigraphy used to test SNTHERM89 temporal sensitivity to initial conditions in Test 4. This stratigraphy was excavated by C. Benson on June 27, 1955 at a site in western Greenland with the elevation of 2918 meters.

Several pits were excavated (146) during the traverse. Yet only three strata were sampled from this stratigraphy database derived from locations on the ice sheet that represent a range in firn conditions during the 4-year period (Test 2 (Figure 7), Test 3 (Figure 8), and Test 4 (Figure 9)).

**Figure 10.**
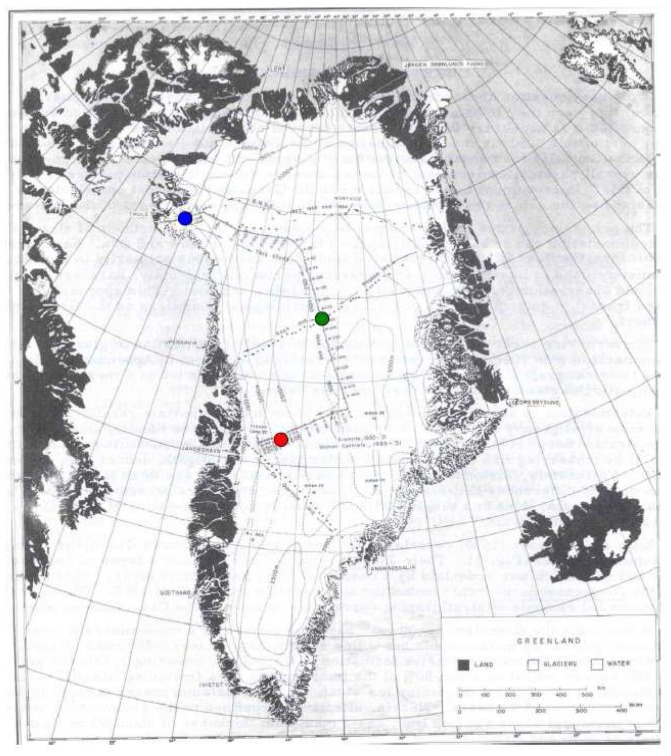
Location of stations where stratigraphy for Test 2 (blue), Test 3 (red), and Test 4 (green) were excavated. (Source: Benson, 1962).

**Figure 11.**
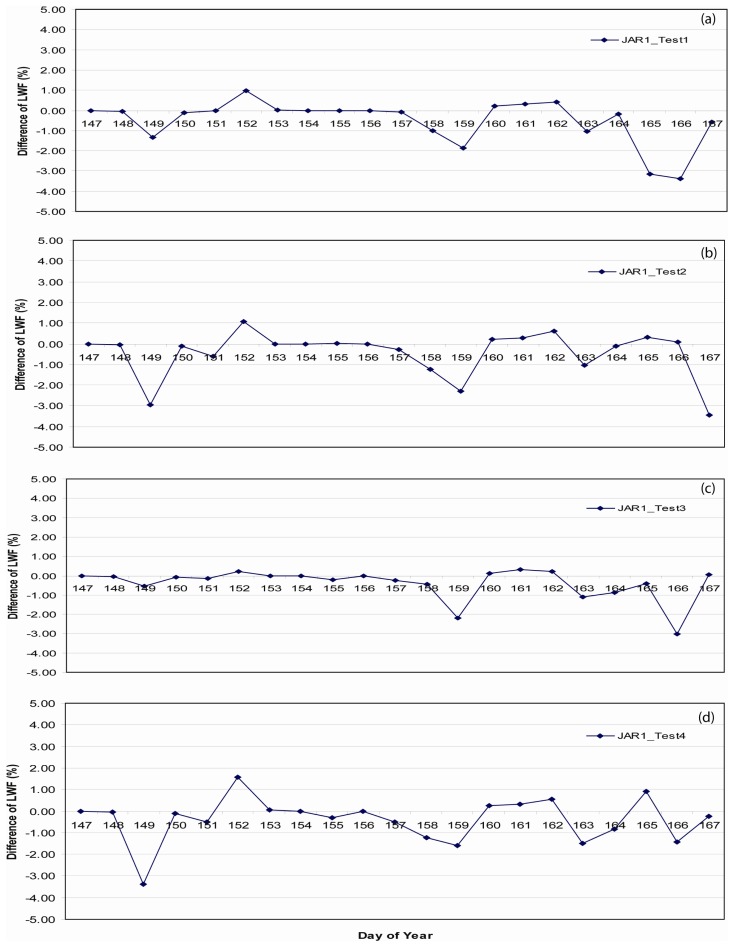
Difference between liquid water fractions (LWF) from upper 5 cm of simulated snow pack derived from SNTHERM89 using stratigraphy from Swiss camp and the test stratigraphy, composited over May 26 to Jun 16 for JAR1.

**Figure 12.**
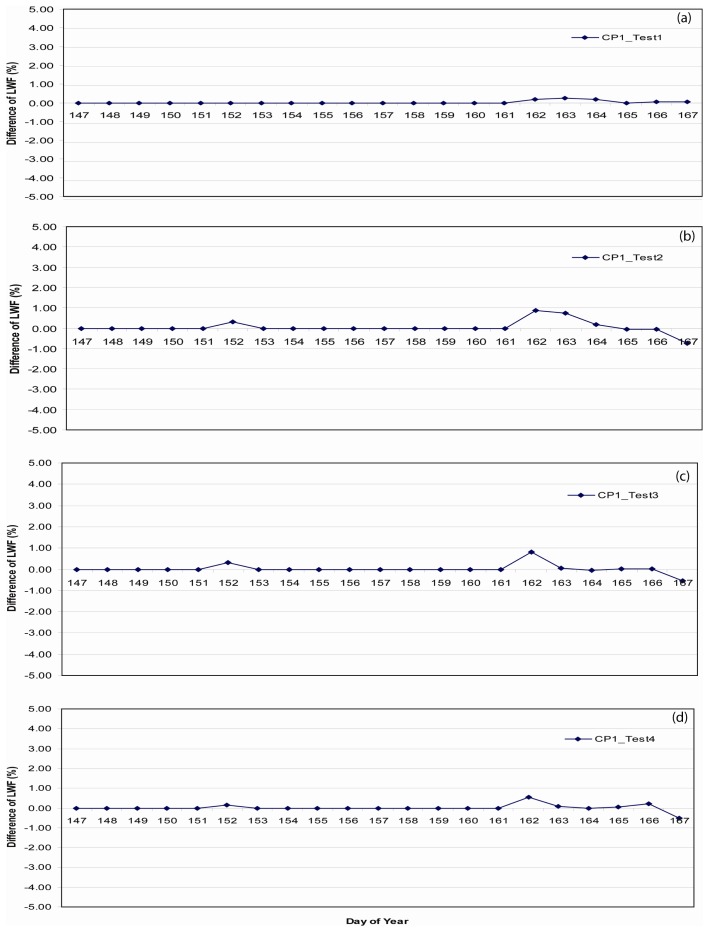
Difference between liquid water fractions (LWF) from upper 5 cm of simulated snow pack derived from SNTHERM89 using stratigraphy from Swiss camp and the test stratigraphy, composited over May 26 to Jun 16 for CP1.

Figure 10 depicts the location of these sample strata. Differences in LWF in the upper 5 cm of SNTHERM89 output derived from runs initialized with ETH/CU stratigraphy versus the test strata, at JAR1 and CP1 were approximately zero (Figure 11, and Figure 12).

## 4. Results

**Figure 13.**
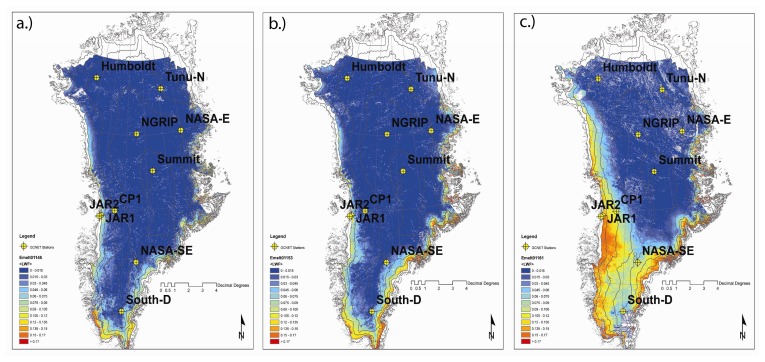
Melt intensity over the Greenland Ice Sheet for composite periods (a) 145 (May 25 - June 1), (b) 153 (June 2 - June 9), and (c) 161 (June 10 - June 17) during the 2001 summer season. Location of GCNET meteorological stations (in yellow) used in this analysis.

E-melt for composite periods 145, 153, and 161 was estimated using MODIS derived surface reflectance and temperature grids as input into the linear empirical inversion model. Figure 13 (a-c) depict maps of E-melt for periods 145 (May 25 - June 1), 153 (June 2 - June 9), and 161 (June 10 -June 17). E-melt maps display the spatial variability in surface melt magnitude patterns and indicate an increasing extent in melt amount from late spring through early summer. Variations in melt magnitude appear to be constrained by elevation.

**Figure 14.**
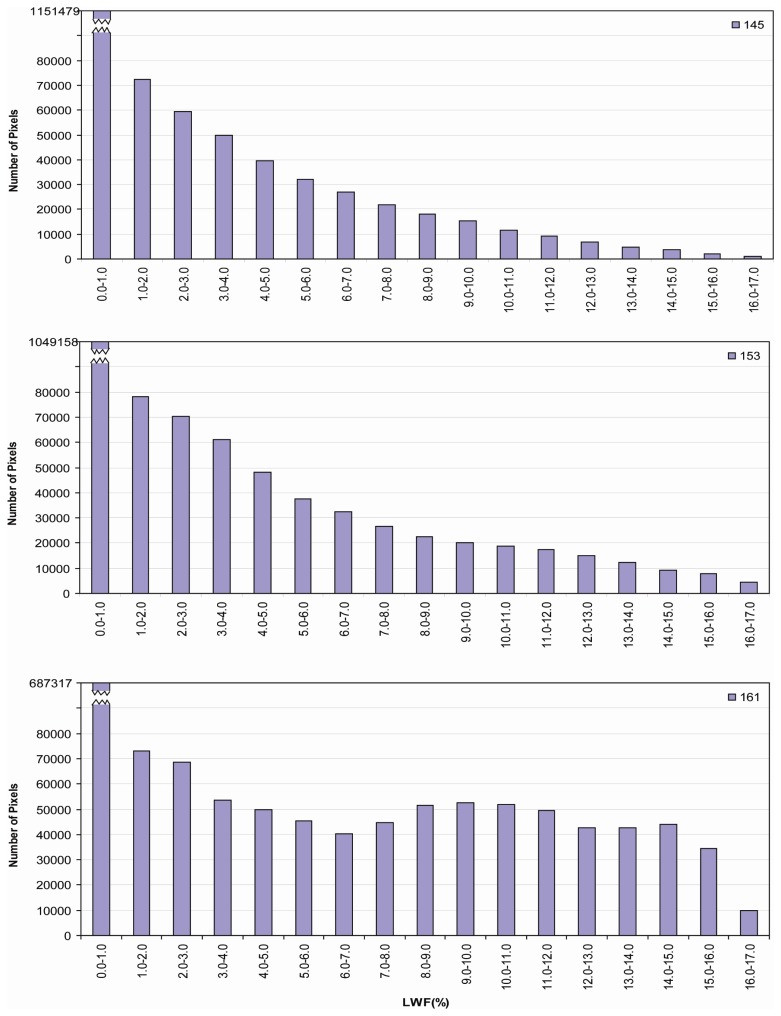
Histograms of effective melt intensity derived from Model I for composite periods 145 (May 25 - June 1), 153 (June 2 - June 9), and 161 (June 10 - June 17) over Greenland ice sheet in 2001.

Higher elevation regions experienced lower magnitudes of LWF as evident by consistently low LWF estimates at Summit and South Dome stations. Lower elevation regions experienced the highest E-melt estimates with a strong latitudinal gradient in intensity from north to south. White spots on the maps indicate cloud cover, which were extracted from MODIS 8-day surface reflectance 500m QA (Quality Assessment) data sets. Histograms (Figure 14) reveal the number of pixels of LWF classified in 1% increment bins for composite periods 145, 153, and 161 and shift to higher fractions from May through June.

### 4.1 E-melt Model Validation and Sensitivity

Evaluation of melt retrieval performance involved comparison of MODIS derived E-melt to those estimated from SNTHERM89 at other GC-NET stations (Table 6), not used to develop the E-melt retrieval model. Additionally, E-melt estimates were compared to scatterometer derived maps of wet and dry firn zones. SNTHERM89 was executed at each of the point validation stations over composite period 161. This period was used because it had higher LWF compared with the other two composite periods (145 and 153).

**Figure 15.**
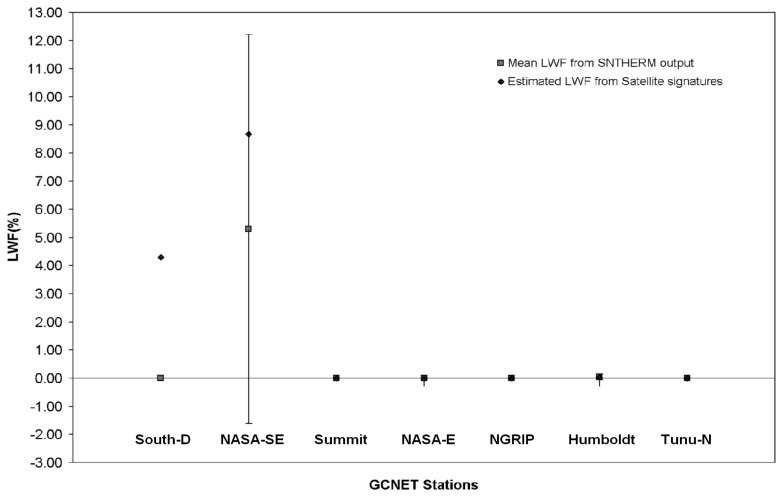
Comparison between mean LWF from SNTHERM89 output and estimated LWF from Model I for the point validation GCNET stations for composite period 161.

Mean LWF was calculated over this composite period from SNTHERM89 outputs in the upper 5cm using Equation [5]. Standard deviation of LWF for the eight day composite period was calculated for each site. SNTHERM89 derived LWF estimated at the point validation sites were compared with satellite derived E-melt (Figure 15). E-melt estimates tend to fall within the variance for each station corresponding to mean LWF derived from SNTHERM89 except station South-D, where E-melt significantly overestimates LWF.

**Figure 16.**
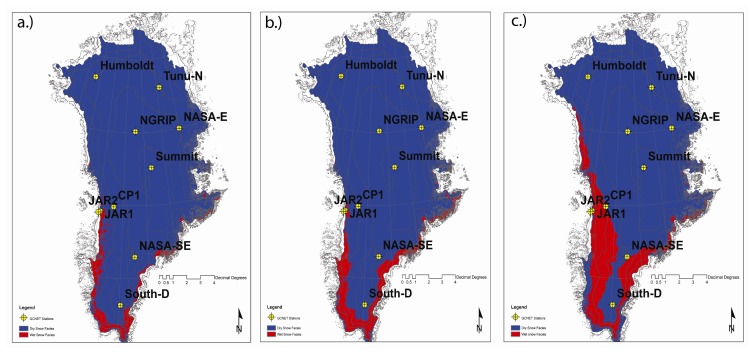
Wet and Dry snow facies on the Greenland ice sheet detected from QSCAT for composite periods (a) 145 (May 25 - June 1), (b) 153 (June 2 - June 9), and (c) 161 (June 10 - June 17) during the summer season. Wet snow zones were determined by a diurnal backscatter change larger than 1.8dB. Dry snow zones were mapped when the diurnal backscatter change was smaller than 1.8dB.

An 8-day composite QSCAT scene was produced by averaging backscatter values of daily QSCAT products for the same composite period of MODIS products. Diurnal backscatter change was calculated for each composite period (Figure 16).

**Figure 17.**
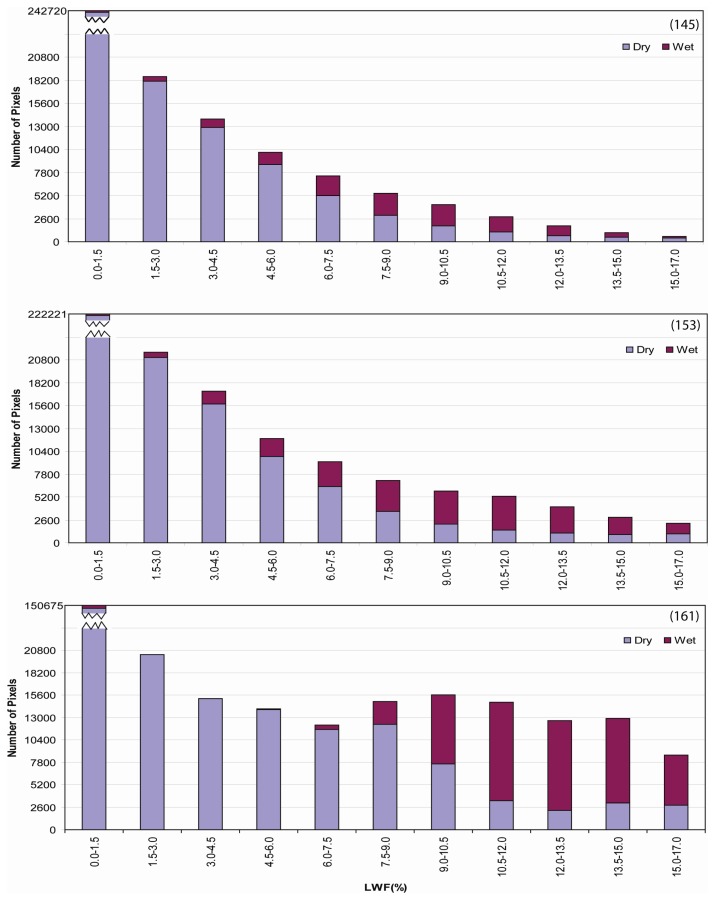
Comparison of E-melt with QSCAT-derived snow facies zones for composite periods 145, 153, and 161.

E-melt maps were resampled to 2.225km in spatial resolution, and compared with the QSCAT-derived wet and dry snow zones. A 1000 meter contour was used to mask sample pixels from both the MODIS and QSCAT images. The 1000 meter contour contains more than 99% of the ice sheet and is sufficient to separate the ice from the rocky coast. Histograms representing dry and wet conditions for each class of MODIS-derived E-melt (Figure 17) display results for all three composite periods. As the summer advances, the distribution of wet zones shifts to higher E-melt magnitudes. Later in the summer (over composite period 161) the distribution of wet snow zones largely corresponds to higher E-melt values.

The E-melt model is solely contingent on MODIS derived 8-day composite, surface temperature and SWIR reflectance as independent variables. MODIS SWIR reflectance products have an estimated relative error of approximately ± 2% (http://modisgsfc.nasa.gov/data/atbd/atbd_mod08.pdf), with typical values less than 5% (Liang et al., 2002). Examination of MODIS LST products through a comparison with automatic weather station temperatures on the GIS, generally indicate fairly reasonable accuracy (1 < 1 °C), but can be as high as 2° (Wan et al., 2002; Hall et al., 2004). We evaluate the impact of MODIS 8-day surface LST and reflectance product accuracy on E-melt retrievals by adding error bias to these variables individually (± 2% for reflectance and ± 1° C for LST) and assess combined error on E-melt magnitudes for early in the melt season (composite period 145) and later (composite period 161). The impact of error biased MODIS products were separately evaluated by inputting maximum positive and negative error grids into the linear retrieval model while holding the other variable fixed to unbiased (measured reflectance or LST) values. This produces estimates of E-melt error when reflectance varies from positive to negative 2% bias while LST is input as unbiased and vice versa for LST averaged over the entire ice sheet.

Therefore, accuracy estimates on E-melt retrievals based on error in reflectance of was ∼0.1% and ∼0.4% based on error in LST for early in the melt season, with combined accuracy of ∼0.7% (reflectance and LST). For later in the melt season, E-melt accuracy based on reflectance is ∼0.2% and ∼1.1% and as high as 2% for LST with combined accuracy as high as ∼2%. These errors represent a first order assessment of the maximum error based on accuracy of input variables.

## 5. Discussion and Conclusion

E-melt retrievals are heavily contingent on the accuracy of both MODIS derived surface reflectance and temperature products. An assessment of the maximum impact of accuracy from these products indicates relatively low errors. The errors increase from the earlier part of the melt season when the magnitude of melt is low to the warmer part of the season where the melt amount is higher. Additionally, errors due to the accuracy of the LST product are larger and become more important later in the melt season. Though late melt season variability in temperatures is lower than the early part of the season, the greater influence temperature has in E-melt retrievals later in the season are likely due to the fact that as surface temperature approach the melting point, significant error in LST can make the difference between melt and no melt conditions. Also, the relationship among temperature, reflectance and melt production can vary throughout the season. Early in the season, the correlation between surface reflectance and temperature can be high (∼ -0.8) at lower elevation and diminishes later in the season due more sustained negative sensible heat flux that stabilize and reduce temperature variability relative to surface albedo (Veenhuis, 2006). Our linear retrieval model does not account for this dynamic and may exhibit seasonally dependent performance.

Point validation analysis, using meteorological data from GC-NET stations that were not used in the E-melt algorithm, indicates relatively good performance with E-melt retrievals within one standard deviation of the mean LWF derived from SNTHERM89 outputs for each point validation station except the South-D station. E-melt overestimated LWF at South-D by 4%. During the 161 period South-D demonstrates very low LWF and stable conditions with little variability. E-melt performed quite well at other stations that demonstrated low variability during the 8-day composite periods and very low mean LWF (approximately zero), therefore, retrieval performance at South-D could be the result of potentially contaminated pixels given the South-D station is bordering a region heavily obscured by cloud cover. Additionally, this bias might be due to the accuracy of the MODIS LST product.

High E-melt magnitudes tend to correspond to QSCAT wet zones, where the highest percentage of wet zones occupy between 10.5-12.0% LWF in composite period 145, and shift to 12.0-13.5% in composite period 153 and 161. Changes in the spatial patterns in wet and dry snow zones throughout the melt season show comparable changes in E-melt although some inconsistencies are apparent. There are dry snow zones near the ice sheet margin that do not correspond to low E-melt values. This may not be solely related to problems with the E-melt algorithm. This inconsistency may be attributed to how radiative information from the optical/thermal parts of the EM spectrum differs from the microwave region. Microwave derived backscatter radiation can be dominated by deeper ice structures such as lenses, pipes, and layers (Baumgartner et al., 1999), while surface temperatures are heavily contingent on surface emission derived from no deeper than several centimeters. Additionally, backscatter can be dominated by subsurface depth hoar and coarse-grain firn (Jezek et al., 1994). Ku-band scatterometers are very sensitive to increased wetness in firn surface layers, resulting in masking backscattering from deeper layers (Nghiem et al., 2001). Therefore, wet snow zones corresponding to lower temperatures (Figure 16) are likely indicative of subsurface melt. Performance issues in the E-melt retrieval may also be related to how the dynamic melt process was simulated in SNTHERM89. Though our model was initialized with a ‘glacier ice’ substrate with snow layers overlying, the melt infiltration and runoff process is not adequately represented in such simulations. SNTHERM89 does not have the capacity to partition melt into run and infiltration and has no specification for preferential flow paths characteristic of melt infiltration in nature.

This retrieval scheme does not take into account melt production during cloud covered periods where downward cloud-forced radiation may be significant in amplifying melt. To assess the impact of clouds on melt a more comprehensive analysis will have to be implemented and is currently under analysis through the use of mesoscale atmospheric model estimates of surface net cloud forced radiation and its relationship to melt extent and occurrence derived from passive microwave systems.

The strong relationship between melt production and surface temperatures may indicate that LST alone could be a suitable variable for this retrieval approach. Retrieval models using LST and reflectance as independent variables singularly were explored and it was determined that the model using coupled optical/thermal independent variables out performed those models that used only a single variable (Peng, 2007).

This novel empirical retrieval scheme can provide valuable information about the spatiotemporal variability of surface melt dynamics that have a significant impact on the mass balance of the GIS. Assessments of melt extent have been informative but have failed to provide vital knowledge amount the amount of melt. This nascent approach can fulfill this need with additional work on validation and refinement. Comparison to traditional passive microwave melt extent and number of melt days will help provide insight into E-melt retrieval performance in addition to exploring application of this retrieval scheme to other years within the MODIS data archive.

## Figures and Tables

**Table 1.** Greenland Climate Network Automatic Weather Station Instruments

Parameter	Instrument	Instrument Accuracy	Sample Interval
Air temperature	Vaisala CS-500	0.1°C	60 s*, 15 s
Air temperature	Type-E Thermocouple	0.1°C	60 s*, 5 s
Relative humidity	Vaisala Intercap	5%<90%, 10%>90%	60 s
Wind speed *	RM Young Propeller-type Vane	0.1m s^-1^	60 s*, 15 s
Wind direction	RM Young	5°	60 s
Station pressure	Vaisala PTB 101B	0.1mb	60 min
Surface height change	Campbell SR-50	1mm	10 min
Shortwave radiation	Li Core SI Photodiode	5-15%	15 s
Net radiation	REBS Q7	5-50%	15 s
Snow temperature	Type-T Thermocouple	0.1°	15 s
Data logger	Campbell Scientific 10X		
Multiplexer	Campbell Scientific Am25T		
GPS	Garmin	1s	1day
Solar panel	Campbell Scientific 20 w		

Sample was taken each 15 s after 1999 site visit except NGRIP AWS. (Steffen and Box, 2001)

**Table 2.** GC-Net Stations used in Calibration Modeling Phase

Station Name	Latitude and Longitude	Altitude (m)
Crawford Point 1	69.8819N, 46.9736W	2022
JAR1	69.4984N, 49.6816W	962
JAR2	69.4200N, 50.0575W	568

**Table 3.** MODIS reflectance and temperature samples extracted to build the empirical model

Reflectance	Temperature (K)	Liquid Water Fraction (%)
0.5887	263.02	0.32
0.4681	260.80	0.00
0.2987	268.06	7.39
0.3665	267.48	5.51
0.3763	268.74	3.94
0.1492	271.76	14.28
0.2325	271.90	16.14
0.3029	271.50	15.65
0.1157	272.52	16.42

**Table 4.** Linear empirical model parameters used to derive effective melt intensity

Model	Dependent Variable	Independent Variable	Coefficient of Reflectance	Coefficient of Temperature	Constant
I	<LWF>	Reflectance, Temperature	-0.136	0.011	-2.822

**Table 5.** Summary of test strata used to evaluate SNTHERM89 sensitivity to initial conditions (data below is for upper 10-12cm within effective radiative zones that contribute to reflectance and temperature).

Strata	Description	Mean Temperature (°K)	Mean Density (kg/m^3^)	Mean Grain Size (m)
Swiss Camp (SC)	Stratigraphy excavated at Swiss Camp on May 16, 2001 at an elevation of 1149 m	263.3	174.3	0.0003
Test 1	Fabricated strata with fewer layers, higher temp gradient and larger change in grain size near surface than Swiss Camp stratigraphy	264.8	107.0	0.0002
Test 2*	Low temperatures, greater range in grain size at depth, and higher densities near top and lower near bottom relative to SC from firn at lower elevation (∼1000m) near the north-eastern coast	259.3	250	0.0005
Test 3*	Inverted near surface gradient, similar density profile as Test 2, most stratified and derived from firn in the percolation zone (∼2200m)	258.0	250.0	0.001
Test 4*	A near linear temperature gradient, derived from firn in the accumulation zone of the ice sheet at an elevation of ∼2800 m	261.2	263.3	0.0005

* Strata were borrowed from 1955 traverse data archive (Benson, 1962)

**Table 6.** GC-Net Stations used for Model Validation

Station Name	Latitude and Longitude	Elevation (m)
Humboldt Gl.	78.5266N, 56.8305W	1995
Summit	72.5794N, 38.5042W	3208
Tunu-N	78.0168N, 33.9939W	2020
NASA-E	75.0000N, 29.9997W	2631
NGRIP	75.0998N, 42.3326W	2950
South-D	63.1489N, 44.8167W	2922
NASA-SE	66.4797N, 42.5002W	2579
